# Supplementary material for: Restricted Access to Working Memory Does Not Prevent Cumulative Score Improvement in a Cultural Evolution Task
Source: Entropy (Basel). 2022 Feb 24;24(3):325. doi: 10.3390/e24030325 (PMC8947658; doi:10.3390/e24030325)
Supplement: Supplementary file 1 [file entropy-24-00325-s001.zip › entropy-1468174-supplementary.pdf]

Supplementary Information: Additional analyses to check for main task offloading onto the distractor task.

As the concurrent task accuracy was significantly lower in the WM block, additional analyses were run on a subset of the data only including trials in which the distractor task was answered correctly. This was in order to analyse data from trials in which participants were more likely to be actually using their working memory to answer the question, rather than focusing solely on the main task.

If the results from the reduced data set were different from using the full dataset, it may indicate that significant offloading of the main task demands onto the concurrent memory task was occurring. The analysis was run using models with the same fixed and random effects as in the original analysis. Unless otherwise stated, all model results stated below describe the same significant effects and interactions as the full dataset.

The model analysing points scored showed significantly fewer points were scored in the transient cues condition ($b=-0.150$, $SE=0.058$, $t(7839)=-2.61$, $p=.009$) and significantly more points were scored on trials where the hint showed more rewards ($b=0.727$, $SE=0.014$, $t(7839)=52.7$, $p<.001$).

The model analysing outperformance of the information indicated higher levels of outperformance in the visible cues condition ($b=-0.047$, $SE=0.022$, $t(7839)=-2.12$, $p=.034$) and significantly less outperformance with higher rewards in the hint ($b=-0.128$, $SE=0.006$, $t(7839)=-20.8$, $p<.001$). In contrast to the model with the full dataset, the interaction between block condition and number of rewards in the hint was not significant, although it did approach significance ($b=-0.006$, $SE=0.003$, $t(7839)=-1.84$, $p=.066$). Post-hoc analysis of the interaction, however, reveals that there is a still a significant difference between the rate of decline in outperformance between the control and WM blocks. The WM block again shows a faster decline as rewards increase, with increased *under*performance when the information trial shows more rewarded squares ($b=0.006$, $SE=0.002$, $z=2.35$, $p=.019$).

Re-analysis of optimal strategy use showed the EF block had significantly higher correct strategy use than the control block (b=0.091, SE=0.029, t(7839)=3.17, p=.002), and the transient cue condition showed significantly lower correct strategy use than the visible cues condition (b=-0.692, SE=0.191, t(7839)=-3.62, p=<.001). There was a significant interaction between block condition and rewards (b=-0.030, SE=0.009, t(7839)=-3.21, p=.001) and between cue condition and the number of rewards (b=0.127, SE=0.041, t(7839)=3.11, p=.002).

Analysis of the errors made in the grid search task again showed significantly more errors were made in the WM block (b=0.313, SE=0.094, z=3.31, p<.001). As in the full data set, there were significant interactions between error type and block (b=-0.783, SE=0.145, z=-5.40, p<.001), between error type and cue type (b=1.65, SE=0.127, z=13.1, p<.001) and the three way interaction between error type, block condition and cue type (b=0.438, SE=0.180, z=2.43, p=.015).

Analysis of clustering of selections again showed significantly more clustering in the WM block compared to the control block (b=-1.52, SE=0.519, t(7848)=-2.94, p=.003). Selections were significantly less clustered when the information trial showed more rewards and when the information trial was less clustered (p<.001 for both). All interactions remained the same, although some of the effects were more marginal, likely due to the reduced size of the data set. The full dataset showed that cue condition did not significantly impact the clustering of selections. This effect remained in the reduced data set (p=.082).

The metacognition task re-analysis showed significantly higher accuracy on trials with high confidence (b=0.935, SE=0.253, z=3.69, p<.001) and a strong right-side bias (b=0.484, SE=0.102, z=4.76, p<.001).

Given the similarity of these results as compared to the full datasets, any impact from offloading of main task demands to the concurrent working memory task was considered to be minimal.
